# Joint line obliquity after lateral closing-wedge high tibial osteotomy does not adversely affect clinical and radiological outcome: a 5-year follow-up study

**DOI:** 10.1007/s00167-023-07532-7

**Published:** 2023-08-10

**Authors:** Tianshun Xie, Maarten R. Huizinga, Inge van den Akker-Scheek, Hugo C. van der Veen, Reinoud W. Brouwer

**Affiliations:** 1 Department of Orthopaedic Surgery, University of Groningen, University Medical Center Groningen, P.O. Box 30.001, 9700 RB Groningen, The Netherlands; 2 Department of Orthopaedic Surgery, Martini Hospital, Groningen, The Netherlands

**Keywords:** Joint line obliquity, Patient-reported outcome, Osteoarthritis progression, Surgical survival, Propensity score matching, Lateral closing-wedge high tibial osteotomy

## Abstract

**Purpose:**

To analyze the association between change in knee joint line obliquity (KJLO) and patient-reported outcome, radiological progression of osteoarthritis, and surgical survival after lateral closing-wedge high tibial osteotomy (HTO).

**Methods:**

A cohort of 180 patients treated in one single hospital with lateral closing-wedge HTO was examined. KJLO was defined by the medial proximal tibial angle (MPTA). To assess the association between KJLO and patient-reported outcome, radiological progression of osteoarthritis, and surgical survival, patient groups were defined: I, postoperative MPTA < 95.0°; II, postoperative MPTA ≥ 95.0°; A, MPTA change < 8.0°; B, MPTA change ≥ 8.0°. Propensity score matching was used for between-groups (I and II, A and B) covariates matching, including age, gender, preoperative lower limb alignment, preoperative medial joint space width (mJSW), preoperative Western Ontario and McMaster Universities osteoarthritis Index (WOMAC) score, wedge size, and postoperative follow-up time. Patient-reported outcome was assessed by the WOMAC questionnaire, radiological progression of osteoarthritis by mJSW and Kellgren–Lawrence (KL) grade progression (≥ 1) preoperatively and at follow-ups (> 2 years). Failure was defined as revision HTO or conversion to knee arthroplasty.

**Results:**

After propensity score matching, groups I and II contained 58 pairs of patients and groups A and B contained 50 pairs. There were no significant differences in postoperative WOMAC score or surgical failure rate between groups I and II or between groups A and B (*p > *0.05). However, the postoperative mJSW was significantly lower in group I than group II (3.2 ± 1.6 mm vs 3.9 ± 1.8 mm; *p = *0.018) and in group A than group B (3.0 ± 1.7 mm vs 3.7 ± 1.5 mm; *p = *0.040). KL grade progression rate was significantly higher in group I than group II (53.4% vs 29.3%; *p = *0.008) and in group A than group B (56.0% vs 28.0%; *p = *0.005).

**Conclusion:**

Increased KJLO (postoperative MPTA ≥ 95.0°) or MPTA change ≥ 8.0° after lateral closing-wedge HTO does not adversely affect patient-reported outcome, radiological progression of osteoarthritis, or surgical survival at an average 5-year follow-up.

**Level of evidence:**

III, retrospective cohort study.

## Introduction

High tibial osteotomy (HTO) realigns the weight-bearing axis in the lower limb, providing a treatment option for medial knee osteoarthritis associated with varus alignment [31]. Two essential techniques are typically used: medial opening-wedge and lateral closing-wedge HTO [42]. However, every HTO creates a change in knee joint line obliquity (KJLO), and the medial proximal tibial angle (MPTA) can be used to describe the KJLO [11, 20, 37].

There is controversial evidence on the association between postoperative KJLO and patient-reported outcomes following medial opening-wedge HTO. Some studies suggest inferior postoperative patient-reported outcomes with an excessive postoperative KJLO [2, 20, 38], and other studies have found no significant difference in postoperative patient-reported outcomes between excessive and normal postoperative KJLO [10, 37, 40]. Additionally, limited research has explored this relationship after a lateral closing-wedge HTO.

Understanding the link between the change in KJLO and patient-reported outcome, radiological progression of osteoarthritis, and surgical survival is necessary when selecting the appropriate knee osteotomy to treat varus medial knee osteoarthritis. Some studies suggest a double-level osteotomy when a valgus-producing HTO is predicted to result in a postoperative MPTA exceeding 95° [20, 28]. However, this recommendation may not be warranted given the current controversy surrounding the association between postoperative KJLO and patient-reported outcomes. There is limited evidence on the associations between postoperative KJLO and radiological progression of osteoarthritis and surgical survival after HTO, highlighting the need for further research in this area.

The purpose of this study is to analyze the associations between change in KJLO and patient-reported outcome, radiological progression of osteoarthritis, and surgical survival after lateral closing-wedge HTO. Our hypothesis is that patients with excessive postoperative KJLO after lateral closing-wedge HTO will present poorer patient-reported outcomes and higher rates of radiological osteoarthritis progression and surgical failure compared to those with normal postoperative KJLO.

## Materials and methods

### Study design

A secondary analysis of patient data from another paper was conducted [13], screening 298 patients undergoing lateral closing-wedge HTO to treat symptomatic medial knee osteoarthritis with varus alignment. Patients were excluded if they (1) did not complete the Western Ontario and McMaster Universities Osteoarthritis Index (WOMAC) questionnaire at postoperative follow-ups (> 2 years), (2) did not have preoperative or postoperative anteroposterior long-standing radiographs, or (3) had a postoperative anteroposterior long-standing radiograph filmed, but the film time was not within 6–18 months after HTO. After applying these exclusion criteria, a total of 180 patients were included in the analyses.

This study design followed the statement of STrengthening the Reporting of OBservational studies in Epidemiology (STROBE) for cohort studies [46] and was approved by the ethics committee of our hospital (MEC no. 2022–005).

### Lateral closing-wedge HTO

The lateral closing-wedge HTO was performed by a single experienced knee surgeon (RWB), in accordance with the procedure described by Huizinga et al. [13] and van Raaij et al. [44]. The procedure involved making an incision from the tibial tuberosity to the posterior aspect of the fibular head, exposing and snaring the common peroneal nerve, resecting the anterior part of the proximal fibular head, and removing the tibial wedge using a calibrated saw guide (Allopro instrument; Zimmer, Winterthur, Switzerland). Lower limb alignment was then corrected, and the osteotomy was fixated with two staples, accompanied by an anterior compartment fasciotomy. The preoperative planning only focused on the hip–knee–ankle angle (HKA) with the goal of achieving a 4-degree valgus alignment [7]. The mechanical lateral distal femoral angle (mLDFA) and MPTA were not considered in the surgical planning for determining the osteotomy type.

### Patient-reported outcome

Patient-reported outcome was evaluated by the WOMAC score including three subscales (pain, stiffness, physical function) [3]. The WOMAC is a disease-specific questionnaire, commonly used to assess pain, stiffness, and physical function in knee osteoarthritis patients and in patients after knee surgery [9, 22]. The WOMAC score was completed preoperatively and at postoperative follow-ups (> 2 years).

### Radiological measurements

**Fig. 1:**
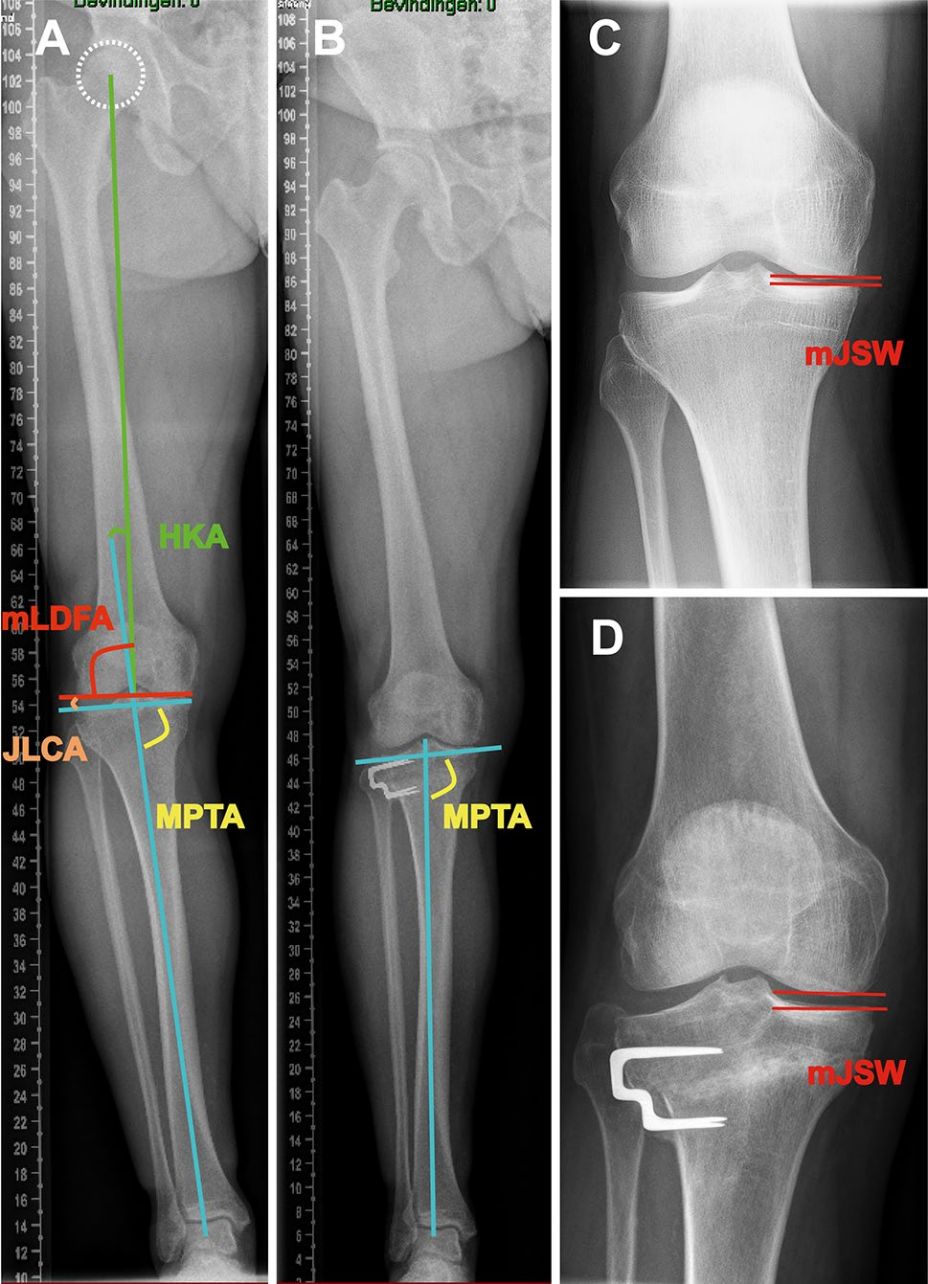
Illustration of radiological measurements. *HKA* hip–knee–ankle angle; *MPTA* medial proximal tibial angle; *mLDFA* mechanical lateral distal femoral angle; *JLCA* joint line convergence angle; *mJSW* medial joint space width

Radiological measurements are illustrated in Fig. 1. The KJLO was defined by the medial proximal tibial angle (MPTA), which is the medial angle between the line tangential to the tibial plateau surface and the tibial mechanical axis [37]. Medial joint space width (mJSW) was measured by the minimum interbone distance between the medial tibial plateau and the medial femoral condyle [39]. HKA was measured by the angle between the femoral mechanical axis and the tibial mechanical axis [6]. The mLDFA was measured by the lateral angle between the tangential line of the femoral condyles and the femoral mechanical axis [32]. Joint line convergence angle (JLCA) was measured by the angle between the tangential line of the femoral condyles and the tangential line of the tibial plateau [32]. Wedge size was obtained by targeting the lower limb mechanical axis at one-third of the lateral knee compartment (4° valgus HKA). The Kellgren and Lawrence (KL) classification was used to grade knee osteoarthritis severity, with four ordinal grades: 1 (doubtful), 2 (mild), 3 (moderate), 4 (severe) [18, 23]. The mJSW and the KL grade progression (≥ 1) were used to evaluate radiological progression of medial knee osteoarthritis [8].

Anteroposterior double-leg standing radiographs were used to assess MPTA, HKA, mLDFA, JLCA, and wedge size, and anteroposterior short knee standing radiographs were used to assess mJSW and KL grade. Patients were positioned with full knee extension and patellar forward during filming. Preoperative and postoperative MPTA, preoperative and postoperative mLDFA, preoperative and postoperative JLCA, and postoperative HKA were measured (TX), and their reliabilities were assessed by two observers (TX, RWB) in 40 patient cases from that patient database, with a three-week interval. The intra-observer and the inter-observer intra-class correlation coefficients of MPTA, mLDFA, JLCA, and HKA were at least good (> 0.75) [24, 48]. Preoperative HKA and wedge size were obtained during planning of lateral closing-wedge HTO (MH). The preoperative and the postoperative mJSW and KL grade were obtained by two orthopedic surgeons who were blinded to the patient’s clinical status using paired reading and sequence-known method [13]. The picture archiving and communication system (Philips Vue, N.V.) was used for radiological measurement, with a minimal determination of 0.01° angle and 0.1 mm distance.

### Surgical failure

Surgical failure was defined as the need for revision HTO or conversion to knee arthroplasty by the time of postoperative follow-up.

### Patient grouping and propensity score matching

Included patients were categorized into two groups based on MPTA cut-off points of postoperative 95° and change of 8°, respectively. These cut-off points were determined from previous biomechanical research, indicating significant shear stress increase and contact stress redistribution beyond these values [28, 47]. Group I: postoperative MPTA < 95.0°; II: postoperative MPTA ≥ 95.0°. Group A: MPTA change < 8.0°; B: MPTA change ≥ 8.0°. The propensity score matching (PSM) method was used to match the covariates between groups I and II and between groups A and B. The present study defined covariates as patient age at surgery, gender, preoperative HKA, preoperative mJSW, preoperative WOMAC (pain, stiffness, and physical function subscores, and total score), wedge size, and postoperative follow-up time [12, 17, 43, 45].

### Sample size calculation

The minimal clinically important difference of WOMAC (a total score difference of 16.1 points) was used to calculate the required sample size [22]. Forty-four patients were needed in each patient group to obtain an effect size of 0.80, an alpha of 0.05, and a power of 0.95 as determined by the Mann–Whitney U test (G*Power software version 3.1.9.7).

### Statistical analysis

SPSS software (version 25) was used for statistical analysis. Distribution of continuous data was checked using the Shapiro–Wilk test and Q–Q plot. PSM was performed with a match tolerance of 0.02. Pearson chi-square tests were used for between-groups comparison of gender and KL grade progression (≥ 1). Fisher’s exact test was used for between-groups comparison of surgical failure rates. Independent t-tests were used for between-groups comparison of parametric continuous data (preoperative and postoperative mJSW, and mJSW change), and Mann–Whitney *U* tests for between-group comparison of non-parametric continuous data (age at surgery, preoperative and postoperative HKA, preoperative and postoperative MPTA, MPTA change, preoperative and postoperative mLDFA, preoperative and postoperative JLCA, preoperative and postoperative WOMAC scores, wedge size, and postoperative follow-up time) and ordinal data (preoperative and postoperative KL grade). The WOMAC score was transformed to a 0–100-point scale where 0 indicates the best possible outcome. A *p < *0.05 was considered statistically significant.

## Results

**Fig. 2:**
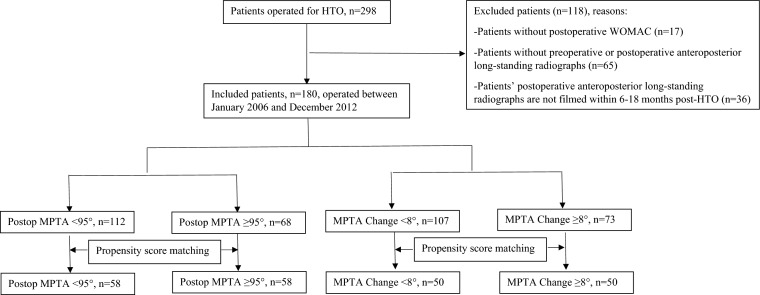
Patient selection process

Patient selection process is depicted in Fig. 2. The baseline characteristic of included patients is presented in Table 1. Of the 180 patients included, postoperative MPTA ranges from 86.1° to 103.1° and MPTA change ranges from 1.4° to 15.3°.

**Table 1:** Baseline characteristics before propensity score matching

Patient baseline characteristics	
Total number of patients	180
Age at surgery, years	51.5 ± 7.6 (24–69)
Gender, male/female, *n* (%)	122/58 (68%/32%)
Operated side, left/right, *n* (%)	94/86 (52%/48%)
Preoperative hip–knee–ankle angle, degrees	5.5 ± 2.4 (1–14)
Preoperative medial proximal tibial angle, degrees	87.3 ± 2.3 (79–92)
Wedge size, degrees	9.5 ± 2.1 (3–6)

Data are shown as mean ± standard deviation (range) unless indicated otherwise

After PSM, 58 pairs of patients were in groups I (postoperative MPTA < 95.0°) and II (postoperative MPTA ≥ 95.0°), and 50 pairs were in groups A (MPTA change < 8.0°) and B (MPTA change ≥ 8.0°). The covariates were matched between groups I and II **(**Table 2) and between groups A and B (Table 3). Comparisons of patient-reported outcome, radiological progression of osteoarthritis, and surgical failure rate between groups I and II and between groups A and B are presented in Table 4.

**Table 2:** Propensity score matching between groups I and II

Covariates	Before propensity score matching			After propensity score matching		
	Group I (postoperative MPTA < 95°)	Group II (postoperative MPTA ≥ 95°)	*p* value	Group I (postoperative MPTA < 95°)	Group II (postoperative MPTA ≥ 95°)	*p* value
Age at surgery, years	51.0 ± 7.8	52.2 ± 7.3	0.344^a^	52.3 ± 7.8	52.0 ± 7.6	0.875^a^
Gender (M/F)	79/33	43/25	0.310^c^	40/18	41/17	0.840^c^
Preoperative HKA, degrees	5.4 ± 2.4	5.7 ± 2.5	0.305^a^	5.6 ± 2.2	5.5 ± 2.5	0.819 ^a^
Preoperative mJSW, mm	3.5 ± 1.5	3.3 ± 1.6	0.353^b^	3.4 ± 1.5	3.5 ± 1.6	0.747^b^
Preoperative WOMAC Pain sub-score	52.1 ± 16.3	57.2 ± 15.9	0.025*^a^	55.5 ± 16.5	55.5 ± 16.3	0.863 ^a^
Preoperative WOMAC Stiffness sub-score	48.8 ± 20.7	52.4 ± 17.3	0.176^a^	50.4 ± 22.8	51.1 ± 17.1	0.826^a^
Preoperative WOMAC Physical function sub-score	46.8 ± 17.1	51.3 ± 17.2	0.076^a^	50.6 ± 18.1	49.4 ± 17.2	0.623^a^
Preoperative WOMAC Total score	48.1 ± 16.2	52.6 ± 15.9	0.069^a^	51.6 ± 17.3	50.8 ± 16.0	0.689^a^
Wedge size, degrees	9.2 ± 2.0	9.9 ± 2.2	0.058^a^	9.6 ± 1.9	9.7 ± 2.2	0.995^a^
Postoperative follow-up time, years	5.1 ± 1.8	5.2 ± 1.8	0.827^a^	5.4 ± 1.8	5.2 ± 1.8	0.490^a^

Data are shown as mean ± standard deviation

*MPTA* medial proximal tibial angle; *HKA* hip–knee–ankle angle; *mJSW* medial joint space width; *WOMAC* Western Ontario and McMaster Universities Osteoarthritis Index

*Statistical significance

^a^Mann–Whitney *U* test

^b^Independent *t*-test

^c^Pearson chi-square test

**Table 3:** Propensity score matching between groups A and B

Covariates	Before propensity score matching			After propensity score matching		
	Group A (MPTA Change < 8°)	Group B (MPTA Change ≥ 8°)	*p* value	Group A (MPTA Change < 8°)	Group B (MPTA Change ≥ 8°)	*p* value
Age at surgery, years	51.3 ± 7.9	51.7 ± 7.2	0.911^a^	51.4 ± 6.8	51.8 ± 6.7	0.959^a^
Gender (M/F)	73/34	49/24	0.877^c^	34/16	32/18	0.673^c^
Preoperative HKA, degrees	4.7 ± 2.0	6.7 ± 2.4	< 0.001*^a^	5.7 ± 1.6	5.6 ± 1.7	0.653^a^
Preoperative mJSW, mm	3.6 ± 1.5	3.3 ± 1.5	0.150^b^	3.2 ± 1.6	3.3 ± 1.3	0.740^b^
Preoperative WOMAC Pain sub-score	54.3 ± 14.9	53.8 ± 18.2	0.858^a^	55.1 ± 14.8	54.9 ± 20.1	0.862^a^
Preoperative WOMAC Stiffness sub-score	51.2 ± 19.0	48.6 ± 20.3	0.425^a^	51.3 ± 19.1	51.0 ± 20.5	0.955^a^
Preoperative WOMAC Physical function sub-score	48.9 ± 16.6	47.9 ± 18.2	0.700^a^	50.0 ± 17.3	50.2 ± 19.2	0.953^a^
Preoperative WOMAC total score	50.2 ± 15.5	49.2 ± 17.3	0.596^a^	51.1 ± 16.0	51.2 ± 18.6	0.896^a^
Wedge size, degrees	8.7 ± 1.8	10.6 ± 2.0	< 0.001*^a^	9.7 ± 1.6	9.6 ± 1.5	0.713^a^
Postoperative follow-up time, years	5.3 ± 1.8	4.9 ± 1.7	0.123^a^	5.3 ± 1.8	4.9 ± 1.8	0.311^a^

Data are shown as mean ± standard deviation

*MPTA* medial proximal tibial angle; *HKA* hip–knee–ankle angle; *mJSW* medial joint space width; *WOMAC* Western Ontario and McMaster Universities Osteoarthritis Index

*Statistical significance

^a^Mann–Whitney *U* test

^b^Independent *t*-test

^c^Pearson chi-square test

**Table 4:** Between-groups comparison after propensity score matching

Measurements and outcomes	Group I (postoperative MPTA < 95°)	Group II (postoperative MPTA ≥ 95°)	*p* value	Group A (MPTA change < 8°)	Group B (MPTA change ≥ 8°)	*p* value
Postoperative HKA, degrees	− 1.4 ± 3.2	− 5.2 ± 2.1	< 0.001*^a^	− 0.8 ± 3.2	− 4.9 ± 2.4	< 0.001*^a^
Preoperative mLDFA, degrees	88.6 ± 1.9	89.8 ± 1.8	< 0.001*^a^	89.0 ± 1.9	89.1 ± 2.5	0.454^a^
Postoperative mLDFA, degrees	88.6 ± 2.0	89.4 ± 1.7	0.088^a^	88.9 ± 2.1	88.8 ± 2.2	0.901^a^
Preoperative JLCA, degrees	2.9 ± 1.3	3.0 ± 1.5	0.763^a^	3.3 ± 1.5	3.0 ± 1.5	0.438^a^
Postoperative JLCA, degrees	2.6 ± 1.4	2.4 ± 1.5	0.534^a^	2.9 ± 1.6	2.5 ± 1.3	0.279^a^
Preoperative MPTA, degrees	86.6 ± 2.2	88.4 ± 2.0	< 0.001*^a^	87.3 ± 2.1	87.0 ± 2.5	0.343^a^
Postoperative MPTA, degrees	92.5 ± 2.0	97.2 ± 1.9	< 0.001*^a^	92.4 ± 2.4	96.4 ± 2.7	< 0.001*^a^
MPTA change, degrees	5.8 ± 2.8	8.8 ± 2.2	< 0.001*^a^	5.1 ± 1.8	9.4 ± 1.1	< 0.001*^a^
Postoperative WOMAC Pain sub-score	24.9 ± 18.5	20.2 ± 20.0	0.103^a^	26.0 ± 18.7	19.9 ± 17.3	0.078^a^
Postoperative WOMAC Stiffness sub-score	30.4 ± 22.0	27.6 ± 21.3	0.554^a^	31.5 ± 21.9	30.8 ± 22.3	0.785^a^
Postoperative WOMAC Physical function sub-score	25.0 ± 20.0	19.2 ± 17.2	0.123^a^	25.3 ± 19.4	21.6 ± 18.8	0.381^a^
Postoperative WOMAC Total Score	25.4 ± 19.1	20.1 ± 17.4	0.103^a^	25.9 ± 18.7	22.0 ± 18.2	0.284^a^
Postoperative mJSW, mm	3.2 ± 1.6	3.9 ± 1.8	0.018*^b^	3.0 ± 1.7	3.7 ± 1.5	0.040*^b^
mJSW change, mm	– 0.2 ± 1.1	0.4 ± 1.3	0.005*^b^	− 0.2 ± 1.4	0.4 ± 1.1	0.025*^b^
Preoperative KL grades 1/2/3/4, *n* (%)	18/32/7/1 (31.0/55.2/12.1/1.7)	27/18/11/2 (46.6/31.0/19.0/3.4)	0.462^a^	13/27/8/2 (26.0/54.0/16.0/4.0)	17/23/10/0 (34.0/46.0/20.0/0.0)	0.499^a^
Postoperative KL grades 1/2/3/4, *n* (%)	7/24/25/2 (12.1/41.4/43.1/3.4)	17/23/14/4 (29.3/39.7/24.1/6.9)	0.041*^a^	6/14/25/5 (12.0/28.0/50.0/10.0)	9/25/15/1 (18.0/50.0/30.0/2.0)	0.007*^a^
KL grade progression (≥ 1), *n* (%)	31 (53.4)	17 (29.3)	0.008*^c^	28 (56.0)	14 (28.0)	0.005*^c^
Surgical failure, *n* (%)	2 (3.4)	0 (0)	0.496^d^	2 (4.0)	0 (0)	0.495^d^

Data are shown as mean ± standard deviation; Measurement change = postoperative measurement – preoperative measurement. The two patients who had surgical failure are the same individuals in both groups I and A: one had revision to lateral closing-wedge HTO due to reoccurrence of painful varus malalignment at 4.7 years postoperative follow-up, the other had conversion to total knee arthroplasty due to reoccurrence of medial knee pain (morbid obesity patient, BMI 51) at 4.3 years postoperative follow-up. A positive value of the HKA indicates a varus alignment, while a negative value indicates a valgus alignment

*MPTA* medial proximal tibial angle; *HKA* hip–knee–ankle angle; *mLDFA* mechanical lateral distal femoral angle; *JLCA* joint line convergence angle; *WOMAC* Western Ontario and McMaster Universities Osteoarthritis Index; *mJSW* medial joint space width; *KL* Kellgren–Lawrence

*Statistically significant

^a^Mann–Whitney *U* test

^b^Independent *t*-test

^c^Pearson chi-square test

^d^Fisher’s exact test is used if > 20% of the expected frequencies > 5

There were no significant differences in postoperative WOMAC or surgical failure rate between groups I and II or between groups A and B. Postoperative mJSW was significantly lower in group I than group II, and in group A than group B. Rate of KL grade progression (≥ 1) was significantly higher in group I than group II, and in group A than group B.

## Discussion

The most important finding is that an increased KJLO (postoperative MPTA ≥ 95.0°) or MPTA change ≥ 8.0° does not have a negative impact on patient-reported outcome and surgical survival after an average follow-up of 5 years. Furthermore, this increase appears to slow down radiological progression of medial knee osteoarthritis. These findings reject our hypothesis.

It was previously investigated that increased KJLO causes unfavorable biomechanical changes. A finite element analysis study reported that MPTA > 95° can result in a rapid shear stress rise at the tibial plateau surface [28]. According to the result of a 10-case cadaveric study, a significant increase of contact stress at the medial spine and lateral meniscus is observed when there is an 8° KJLO increase in lateral direction (from 1° to 9° laterally) at both 0° and 20° knee flexion [47]. However, these biomechanical changes did not negatively influence the clinical and radiological results in our patient group 5 years after lateral closing-wedge HTO. A possible explanation is that these biomechanical changes may not be the primary determinants of the clinical and radiological outcomes, and the follow-up length we used may not be long enough to fully observe the effects on these outcomes.

Besides MPTA, other angles are used to assess KJLO, such as joint line orientation angles and the Mikulicz joint line angle [2, 26, 37, 41]. In the present study, MPTA was used, as it is independent of factors, such as osteoarthritis grade, single-leg/double-leg standing position, and stance width during radiograph filming, making it the preferred choice over the other angles [48].

The present study demonstrates that the increased KJLO does not affect patient-reported outcome. This finding aligns with previous studies that used similar but different questionnaires with varying follow-up lengths post-HTO, finding no significant differences in outcomes when comparing patients with postoperative MPTA < 95° and > 95°: Sohn et al. [40] used WOMAC and the Knee Society Score (KSS) with 1-year follow-up; Kim GW et al. [19] used the WOMAC, KSS, and Hospital for Special Surgery knee-rating score with > 4 years of follow-up. Goshima et al. [10] used the Japanese the orthopedic association score, Oxford knee score, and Knee injury and Osteoarthritis Outcome Score (KOOS) with mean postoperative follow-up of 6.1 years; Rosso et al. [37] used the WOMAC and KSS with mean follow-up of 10 years. By contrast, other studies report inferior outcomes that surpass the minimal clinically important difference of the questionnaire when postoperative MPTA > 95°, including Akamatsu et al. [2] with KSS and KOOS at 2-year follow-up, Kim JS et al. [20] with KSS and Short-Form 36 at a mean follow-up of 5.6 years, and Schuster et al. [38] with the International Knee Document Committee subjective knee score at a mean follow-up of 10 years. The present study distinguishes itself by the use of the PSM method to match covariates one-on-one, with a consideration of various covariates that may affect patient-reported outcome measures. Moreover, these studies all investigate medial opening-wedge HTO, whereas the present study analyzes lateral closing-wedge HTO. There are biomechanical differences between postoperative medial opening-wedge and lateral closing-wedge HTOs, such as knee-loading distribution [30], which might contribute to the reported variations in postoperative patient-reported outcome measures.

Patients with increased KJLO appear to maintain the mJSW at follow-ups. It has been reported that mJSW can continuously increase up to 3 years post-HTO [21]. However, the clinical interpretation of mJSW is still under debate. Some suggest it reflects the thickness of the medial knee cartilage [4, 39] or the status of the medial meniscus [14, 16]. The mJSW narrowing is often used to evaluate medial knee osteoarthritis progression [8, 36], whereas post-HTO changes in mJSW may be linked to the weight-bearing line ratio [15, 27]. A lateral closing-wedge HTO causes lateral defect laxity due to a decrease in the height of the lateral tibial plateau. This defect laxity, along with the postoperative valgus alignment, contributes to the increased KJLO. One possible explanation for our results is that patient with a higher increase in KJLO has a more valgus postoperative HKA, along with more significant tibial bony valgisation and increased lateral defect laxity following a lateral closing-wedge HTO, which in turn results in a larger opening of the medial knee compartment. Limited evidence is published on the association between MPTA and mJSW. One study reported that 1° MPTA decrease can significantly increase the odds of mJSW narrowing by 21% in medial knee osteoarthritis patients with a 2-year follow-up [33]; another reported no significant difference in postoperative MPTA (92.7° vs 91.9°) between patients with increased mJSW and decreased mJSW (0.8 mm vs − 0.5 mm) 3 years following medial opening-wedge HTO [21]. A medial opening-wedge HTO can increase medial collateral ligament strain, potentially affecting mJSW if no release technique is used [1, 5, 34]. By contrast, a lateral closing-wedge HTO has minimal impact on the medial collateral ligament [34]. Future studies should investigate the long-term impact of increased KJLO on lateral cartilage and meniscus status post-HTO.

The absence of mJSW narrowing in patients with increased KJLO, as observed in our study, may explain their lower rate of KL grade progression in the medial knee compartment. However, it is important to note that the evaluation of KL grade and mJSW is based on radiographs, which is not an ideal imaging modality for assessing osteoarthritis progression and cartilage thickness. Hence, future studies using magnetic resonance imaging (MRI) or arthroscopy after a long-term follow-up post-HTO are warranted to confirm these findings.

Another important finding of our study is that KJLO increase does not affect surgical failure rate after HTO. Only one other study compared the rate of revision to knee arthroplasty between postoperative MPTA ≤ 95° and > 95° following medial opening-wedge HTO, finding no significant difference over an average 10-year follow-up [38]. Another study found that a postoperative MPTA ≥ 95° can help prevent recurrent varus malalignment following a valgus-producing HTO, as observed at short-term follow-up of 1 year [35]. Likewise, in the present study, surgical failure in one of the two revised patients with MPTA < 95° was due to the reoccurrence of painful varus malalignment. Future studies may explore the impact of increased KJLO on conversion to total knee arthroplasty following a failed HTO, including surgical complexity and choice of tibial component.

To achieve a targeted alignment and prevent under-correction after a valgus-producing HTO, a large postoperative KJLO may be predicted during surgical planning, but lowering it down to the normal range (MPTA, 85°–90°) can be challenging [32]. Based on the present finding, 95° MPTA may not be a strict cut-off point that indicates a double-level osteotomy, and a MPTA change > 8° post-HTO also appears tolerable. Notably, our results do not imply that the postoperative KJLO can be entirely disregarded during HTO planning, as an increase in KJLO can have other negative impacts on gait pattern and knee kinematics [25, 29].

The strength of this study lies in its contribution toward filling the knowledge gap regarding the influences of KJLO on outcomes after a lateral closing-wedge HTO. We used a reliable KJLO measurement method and utilized the PSM method to minimize the influence of unmatched covariates on comparing outcomes. Besides the postoperative KJLO, we also examined the effects of KJLO change.

As a retrospective study, limitations include insufficient assessment of the effects of increased KJLO on knee cartilage and meniscus status. Since mJSW is an indirect indicator for assessing medial knee cartilage and meniscus status, and given the controversy surrounding what it actually represents, MRI or arthroscopy would be more suitable modalities for this assessment. Also, obesity might have negative effects on outcomes and can lead to early HTO failure; however, the data of patient body mass index at surgery was incomplete and could not be used in the analyses.

## Conclusions

Increased KJLO (postoperative MPTA ≥ 95.0°) or MPTA change ≥ 8.0° after lateral closing-wedge HTO does not adversely affect patient-reported outcome, radiological progression of osteoarthritis, or surgical survival at an average 5-year follow-up. The decision to choose a double-level osteotomy over HTO should not be exclusively based on a predicted increase in KJLO (postoperative MPTA ≥ 95.0°) at planning.

## Data Availability

The datasets generated during the current study are available from the corresponding author upon reasonable request.

## Abbreviations

HKA: Hip-knee-ankle angle
HTO: High tibial osteotomy
JLCA: Joint line convergence angle
KJLO: Knee joint line obliquity
KL: Kellgren and Lawrence
KOOS: Knee injury and Osteoarthritis Outcome Score
KSS: Knee Society Score
mJSW: Medial joint space width
mLDFA: Mechanical lateral distal femoral angle
MPTA: Medial proximal tibial angle
PSM: Propensity score matching
WOMAC: Western Ontario and McMaster Universities Osteoarthritis Index
